# Determinants of Missed Games Following Concussions in the National Football League

**DOI:** 10.3389/fspor.2020.595445

**Published:** 2020-11-19

**Authors:** Elizabeth C. Heintz, Lindsey Breaux, Abilgail Lewis, Jeremy J. Foreman

**Affiliations:** School of Kinesiology, University of Louisiana at Lafayette, Lafayette, LA, United States

**Keywords:** American football, injury, NFL, quarterback, return to play

## Abstract

Concussions in the National Football League (NFL) are a rising topic of concern and debate. With the implementation of new protocols, NFL players are required to be removed from play and must follow specific processes before full return to play after sustaining a concussion. While concussion severity is the primary determinant of time-off, it is possible that other factors associated with player value influence time-off as well. The purpose of this study is to examine determinants of NFL games missed after a concussion. NFL concussion data from 2012 to 2015 were used in conjunction with player salary, position, previous concussions, average plays per game, and season during which the concussion occurred. Results indicate that quarterbacks and players who sustained multiple concussions missed more games, while players involved in more plays per game miss less games, providing evidence that player health and value are determinants of time-off after a concussion. Additionally, the number of games missed has increased each year, which could be the result of NFL efforts to increase safety and increased media attention.

## Introduction

Concussions in the National Football League (NFL) are common injuries and a widespread concern. The physically brutal nature of football makes athletes susceptible to concussions and repeat concussions in the NFL (Casson et al., 2011b). After denying the severity and prevalence of concussions in professional football for years (Fainaru-Wada and Fainaru, 2013), the NFL now admits that approximately one-third of NFL players suffer from long-term brain damage from concussions (Belson, 2014).

Although the NFL implemented policies and changes to increase player safety (Gove, 2012), concussions remain common (Casson et al., 2011a,b). In the NFL, there are ~140 concussions reported each season, and it is estimated that at least one player on a team suffers a concussion every five games (Casson et al., 2010). Now, the NFL's Return-to-Participation Protocol requires players to rest after sustaining a concussion (NFL Head, Neck, and Spine Committee, 2018b), often causing them to miss games as they recover. With increased attention on concussions in the media, and as more protocols are put in place, time-off after concussions has increased in the National Basketball Association (NBA) and the National Hockey League (NHL) in recent years (Wennberg and Tator, 2008; Padaki et al., 2016). However, time-off from a concussion varies individually and can last longer than 3 weeks in some cases (Casson et al., 2011a).

While the length of a player's recovery time is often dictated by medical severity (Casson et al., 2011a), it is also possible that other factors influence time-off. In football, certain positions are perceived as being more valuable to the outcome of a game than others (Brooks, 2015), which could impact the number of games players miss. Further, because players may be considered more or less valuable based on their position, factors such as salary and experience could also influence time-off following a concussion. Thus, the purpose of this study is to examine determinants of NFL games missed following a concussion.

A better understanding of what factors influence time-off after a concussion in the NFL could have implications for athletes, managers, and medical personnel. Athletes could increase their awareness of the factors that may impact their risk for returning to play before a full recovery, and both athletes and managers could better understand other underlying influences than merely concussion severity that dictate time-off. Finally, results from this study could assist medical personnel by revealing which athletes are at higher risks of returning to play earlier and could impact increased monitoring of these athletes to ensure they fully recover.

## Literature Review

Although a single concussion may not be life-threatening (Centers for Disease Control Prevention, 2019), repeated head trauma experienced in football is associated with severe repercussions (Abreu et al., 2016). Among the medical community, it is largely believed that there is a link between concussions and chronic traumatic encephalopathy (CTE), which is the buildup of protein in the brain (Mayo Clinic, 2019). CTE is a neurodegenerative disease that causes memory loss, depression, anger, sleep disorders, and suicidal thoughts (Stone, 2017; Mayo Clinic, 2019), and has been found in many former NFL players who experienced repeated head trauma in practices and games (Mayo Clinic, 2019). With the discovery of the prevalence of CTE in professional football players, concussions in football have become a topic of interest in the media, causing spectators to question the safety of the game (Stone, 2017). Further, the suicides of multiple former NFL players led to increased interest in CTE and the dangers of brain injuries in football (Stone, 2017).

While the NFL originally denied that concussions were an issue in professional football (Fainaru-Wada and Fainaru, 2013), it now admits that at least one-third of its players likely suffer from brain damage from repeated concussions (Belson, 2014). One of the earliest surveys on CTE in former NFL players revealed that over half had obtained concussions during their careers and those who had suffered more concussions also experienced more severe neurological disorders later in life (American Academy of Neurology, 2000). Although repeated head trauma in sports has long been a concern of the medical community, the high association of CTE in NFL players has more recently sparked concern in NFL coaches, players, fans, and the media (Solomon, 2018). While the NFL may have denied the risks of professional football, these findings called for change.

With the prevalence of CTE and increased attention from the media on the dangers of football, the NFL has implemented rule changes and concussion protocols in response (Brenson, 2018). Additionally, organizations were founded to research the effects of concussions and to protect players (Brenson, 2018). Further, sideline and in-booth officials, as well as physicians, are responsible for spotting possible concussions that occur during games (Sites, 2017). The NFL Head, Neck, and Spine Committee has also implemented a return-to-participation protocol that requires rest and recovery before an athlete begins low-intensity exercise and eventually full return to play (NFL Head, Neck, and Spine Committee, 2018b).

Another recent addition to the NFL is unaffiliated neurotrauma consultants, who monitor video and audio game replays for potential head, neck, or spine injuries (Mortensen, 2017). “No-Go” criteria that warrant immediate removal from play have also been implemented to reduce risk to players from repeated concussions (NFL Head, Neck, and Spine Committee, 2018a). Finally, players who sustain concussions and undergo concussion evaluation during a game are required to have a follow-up concussion evaluation the next day (NFL Head, Neck, and Spine Committee, 2018b).

Research suggests that tight ends, running backs, wide receivers, and cornerbacks are the positions most likely to sustain concussions (Dai et al., 2018), while there is also evidence that defensive secondaries, kick units, running backs, and linebackers are most likely to suffer multiple concussions (Casson et al., 2011b). Additionally, Dai et al. (2018) found that concussions were more prevalent in the NFL during the second half of the season. Despite that a single concussion does not seem to negatively impact performance if adequate recovery time is allowed, multiple sub-concussive hits could cause decreases in player performance (Reams et al., 2017). Further, previous research has also shown that concussions cause shorter careers, earlier franchise releases, and decreases in salaries for NFL players (Navarro et al., 2017), indicating that concussions have negative health and financial outcomes for players.

Concussion severity and differences in individual recovery times are the primary medical determinants of time-off after a concussion (Casson et al., 2011a). While reported concussions cause most athletes to miss at least 1 week of play, severe cases can keep athletes out for over 3 weeks (Casson et al., 2011a). As the effects of concussions have become better understood and the media increases its focus on concussions in sports, time-off after a concussion has increased in both the NBA (Padaki et al., 2016) and NHL (Wennberg and Tator, 2008). Additionally, NHL athletes who fly on commercial air flights after a concussion tend to miss more games than athletes who do not fly after a concussion, as there is evidence that hypoxia can lead to increased brain dysfunction after a concussion (Milzman et al., 2014). Further, athletes who sustain multiple concussions tend to have more time-off compared to athletes who sustain a concussion for the first time (Casson et al., 2011b; Dai et al., 2018). Kumar et al. (2014) found that age may affect the number of games NFL players miss following a concussion, as younger, less experienced players were more likely to miss at least one game, while older, more experienced players tended to not miss any games. Also, players who suffered a concussion later in the season were likely to return to play without missing a game (Kumar et al., 2014).

It is also possible that other factors besides player health impact time-off after a concussion, such as player contributions to a game or monetary value. Professional athletes are celebrities and brands that generate revenue, giving athletes a commercial value (Carlson and Donavan, 2013; Arai et al., 2014). More recognizable athletes generate more revenue and attract more spectators, increasing their celebrity status (Carlson and Donavan, 2013). Furthermore, fans develop emotional attachments to athletes, making them idols as well (Hyman and Sierra, 2010; Carlson and Donavan, 2013). Thus, professional athletes are more than just athletes; they are sources of revenue and individual brands (Hyman and Sierra, 2010; Carlson and Donavan, 2013; Arai et al., 2014). Therefore, the value of a specific athlete based on revenue generation could be a consideration of the amount of time-off an athlete receives following an injury. Likewise, player value based on audience engagement and emotions could also be a determinant of time-off for an athlete because of their celebrity status.

Additionally, NFL positions have been ranked by value based on the contribution of each position to the outcome of the game (Brooks, 2015). The quarterback is considered the most valuable, followed by other top playmakers such as wide receivers (Brooks, 2015). Because most quarterbacks are right-handed, the left tackle is also considered one of the most valuable positions (Brooks, 2015). With certain positions considered more valuable, position could impact the amount of games a player misses after a concussion. For example, valuable players could have more time-off in order to protect them from further injury. Contrarily, it is also possible that valuable players have less time-off because of their contribution to the game and a team's success. Therefore, this study examines factors of both player health and player value in relation to games missed following a concussion in the NFL.

## Method

A longitudinal design is used to examine the determinants of games missed after sustaining a concussion in the NFL. There was no Institutional Review Board (IRB) approval for this study, as the research did not involve any interaction with human participants or animal subjects and all data used are publicly available. Concussion data were collected from *PBS Frontline's Concussion Watch*, which includes information on officially reported concussions in the NFL. The sample period was from 2012 to 2015 and includes NFL players who missed one or more games after a confirmed concussion and who were included in the NFL's injury report with a concussion or head injury (Breslow, 2012). During this period, 643 concussions were reported. Twenty-six concussions were sustained by quarterbacks, 99 by offensive linemen, and 190 by defensive linemen.

The dependent variable in this study is the number of games missed following a concussion (*GmsMiss*). The independent variables of interest are (a) player salary measured in millions of dollars (*Salary*), (b) whether the player was a quarterback (*QB*), and (c) the average number of plays per game the player was involved in prior to receiving a concussion (*AvgPlays*), all of which are directly related to player value. Players with higher salaries and those involved in more plays per game likely have greater impacts on game outcome and are considered more valuable to the team; therefore, the number of games missed by these players is likely different than players with lower salaries or those less involved. Likewise, as quarterbacks are considered the most valuable position on a team (Brooks, 2015), this likely impacts the amount of time-off they receive following a concussion.

Three more variables that could impact the number of games missed following a concussion are included in the model. As time-off tends to increase with each successive concussion (Casson et al., 2011b; Dai et al., 2018), the number of concussions previously sustained during the sample period (*PrevConc*) was included. Additionally, as the amount of time-off following a concussion has increased over recent years in other professional sports (Wennberg and Tator, 2008; Padaki et al., 2016), the season during which the concussion occurred (*SeasCnt*) and the week of the season the injury occurred (*Week*) are included in the analysis. Ordinary least squares (OLS) regression analysis is used to estimate the effects of the independent and control variables on the number of games missed after a concussion to determine predictors of games missed in the NFL following a concussion. OLS regression reports *p*-values to determine statistically significant relationships and coefficients to estimate the size of relationships between variables that can be used to estimate the effects of the independent variables on the dependent variable.

## Results

The mean games missed following a concussion was 0.99 and ranged from 0 to 13 games. Four percent of the sample included quarterbacks, and the mean number of plays that players averaged was 47.32 with a standard deviation of 20.04. Players in the sample had between zero and three previously reported concussions (Table 1).

**Table 1:** Variable descriptions and descriptive statistics.

**Variable**	**Description**	**Mean**	**Std. Dev**.	**Min**.	**Max**.
GmsMiss	# of games missed after sustaining a concussion	0.99	1.57	0	13
Salary	Player salary in millions for the season	2.40	3.31	0.08	35.26
QB	Dummy variable (1 if quarterback, 0 if not)	0.04 (4%)	0.19	0	1
AvgPlays	Average # of plays/game before concussion	47.32	20.04	0	85
PrevConc	# of concussions a player previously sustained	0.14	0.40	0	3
SeasCnt	Season count from 2012 (2012 = 1, …, 2015 = 4)	2.60	1.17	1	4
Week	Week count from week 4 through week 17	10	4.34	4	17

Although player salary did not significantly impact games missed after a concussion, other indicators of player value, such as player position and the average number of plays a player was involved per game, significantly affected time-off after a concussion (Table 2). In this sample, quarterbacks missed approximately one more game than players in other positions (*p* = 0.003). Contrarily, players who averaged more plays per game had less time-off following concussions (*p* = 0.033). For example, a player averaging 27 plays per game (i.e., one standard deviation below the mean) is expected to miss ~0.28 more games following a concussion than a player averaging 67 plays per game (i.e., one standard deviation above the mean).

**Table 2:** OLS regression estimates for games missed following a concussion.

**Variable**	**Coefficient**	**Standard Error**	***p*-Value**
Salary	−0.003	0.023	0.897
QB	0.908^***^	0.284	0.003
AvgPlays	−0.007^**^	0.003	0.033
PrevConc	0.252^*^	0.129	0.059
SeasCnt	0.163^*^	0.080	0.050
Week	−0.011	0.016	0.470
Constant	0.945^***^	0.313	0.005

* *p < 0.1*,

** *p < 0.05*,

*** *p < 0.01*.

Results from this regression also indicate that player health and season play a role in games missed following a concussion. Previous concussions significantly increased games missed, with each concussion a player sustained increasing the number of games missed by ~0.25 (*p* = 0.059). The season during which a concussion was sustained also affected the amount of time-off a player received, as players missed an average of 0.16 more games each subsequent year (*p* = 0.050). However, the week of the season during which a player received a concussion did not significantly affect the number of games missed. We are confident in the results of the analysis given that the variance inflation factors (VIFs) were no higher than 1.15, the correlation coefficients did not exceed 0.314, and a stepwise regression revealed that the same variables were statistically significant that the full model found. The VIFs and correlation coefficients can be found in Table 3, and the stepwise regression results can be found in Table 4.

**Table 3:** Variable correlations and variance inflation factors.

**Variable**	**VIF**	**GmsMiss**	**Salary**	**QB**	**AvgPlays**	**PrevConc**	**SeasCnt**
Salary	1.15	−0.008					
QB	1.14	0.093	0.188				
AvgPlays	1.04	−0.076	0.314	0.072			
PrevConc	1.03	0.059	0.001	−0.042	0.135		
SeasCnt	1.02	0.125	0.034	−0.047	−0.037	0.096	
Week	1.01	−0.025	−0.044	−0.014	−0.081	0.012	−0.000

**Table 4:** Stepwise regression results for games missed following a concussion.

	**Step 1**		**Step 2**		**Step 3**		**Step 4**		**Step 5**		**Step 6**		**Final Model**	
**Variable**	**Coef**.	***p***	**Coef**.	***p***	**Coef**.	***p***	**Coef**.	***p***	**Coef**.	***p***	**Coef**.	***p***	**Coef**.	***p***
Salary	<0.01	0.87	–	–	–	–	–	–	–	–	–	–	–	–
QB			0.79	0.02	0.83	0.02	0.86	0.01	0.90	<0.01	0.90	<0.01	0.90	<0.01
AvgPlays					−0.01	<0.01	−0.01	0.04	−0.01	0.04	−0.01	0.04	−0.01	0.04
PrevConc							0.30	0.03	0.25	0.06	0.25	0.06	0.25	0.06
SeasCnt									0.16	0.05	0.16	0.05	0.16	0.05
Week											−0.01	0.47	–	–
Constant	1.00	<0.01	0.96	<0.01	1.26	<0.01	1.26	<0.01	0.82	<0.01	0.95	<0.01	0.82	<0.01

## Discussion

The purpose of this study was to analyze determinants of games missed for NFL players after a concussion. Results from this study indicate that quarterbacks and players who sustain multiple concussions miss more games after a concussion, while players involved in more plays per game receive less time-off after a concussion. Time-off after a concussion has also increased by NFL season. Therefore, time-off following concussions is determined by player value, player health, and time-based factors. Furthermore, although the NFL has taken steps to increase player safety, new information regarding concussions and their effects are constantly being discovered; therefore, these protocols should continue to be evaluated to ensure player safety.

### Player Value and Return to Play

While the NFL's Return-to-Participation Protocol calls for all players to receive a minimum amount of care following a concussion (NFL Head, Neck, and Spine Committee, 2018b), the results of the present study suggest that a disparity exists regarding time-off following a concussion. More specifically, a player's value appears to impact the number of games missed after a concussion in the NFL. Though salary did not have a statistically significant effect on the number of games missed, quarterbacks, who are often considered the most valuable position on a team (Brooks, 2015), received significantly more time-off after a concussion than other players. Quarterbacks are typically viewed as the team leader and decision-maker (Brooks, 2015). Additionally, quarterbacks are valued as being the main contributors to scoring (Hoffer and Pincin, 2019). Furthermore, Mulholland and Jensen (2019) found that a team's best investment is their quarterback. This suggests that quarterbacks are highly valued for their contributions to game outcomes; thus, they may get more time-off after a concussion because coaches and managers want to ensure that they can perform at optimal levels upon their return. It is also possible that attention devoted to quarterbacks during games, such as multiple television cameras constantly focused on quarterbacks throughout a game, may influence decisions regarding time-off following concussions since millions of television viewers likely witnessed the concussion. Nevertheless, this disparity in time-off between quarterbacks and other positions could allow quarterbacks to recover more from a concussion compared to other players.

In contrast, players involved in more plays (i.e., players more heavily relied upon on a regular basis) received less time-off following a concussion. These results indicate that while quarterbacks are typically protected and allowed more time-off, other players are rushed back to play sooner than players who are not as heavily relied on. These heavily used players may be rushed back from a concussion because they have responsibilities such as protecting the quarterback or because they are not as valued for their cognitive abilities (Heintz et al., in press). Compared to other offensive positions, quarterbacks have significant decreases in performance in the season following their return from a concussion (Navarro et al., 2017; Jildeh et al., 2019; Heintz et al., in press), which is likely indicative of changes in neurocognitive abilities. Thus, it is possible that players in other positions have changes in neurocognitive performance following a concussion, but these changes are more pronounced in quarterbacks who rely more heavily on cognitive abilities (Heintz et al., in press). With such decreases in performance less obvious in non-quarterbacks, heavily relied on players could return to play before they are fully recovered. Further, as repeated head trauma leads to more severe neurological consequences compared to a single concussion (American Academy of Neurology, 2000) and repeat concussions are more likely to occur prior to full recovery (Guskiewicz et al., 2003), players involved in more plays may be at higher risk for long-term brain damage and CTE due to less time-off after a concussion. Therefore, it appears that player value does affect the number of games NFL players miss following a concussion, although in different ways for different positions based on how the players are valued.

### Player Health and Return to Play

Although Kumar et al. (2014) did not find evidence of prior concussions influencing time-off following a subsequent concussion, consistent with previous research [e.g., Casson et al. (2011b) and Dai et al. (2018)], results from the present study show that the number of games missed following a concussion increases with each successive concussion a player suffers. Therefore, in addition to player value, player health is a factor considered when determining when an athlete should return to play. The discrepancy between Kumar et al.'s study and the present study may result from the time periods analyzed, given that Kumar did not find a relationship between previous concussions sustained and games missed following a subsequent concussion in the sample period 2008–2012; however, in this more recent sample period (i.e., 2012–2015), successive concussions appear to play a role in determining time-off following a concussion. This could be the result of increases in the NFL and medical community's awareness for the risk of developing CTE with multiple concussions (Mayo Clinic, 2019), media attention and public awareness (Martin et al., 2017), or preventative efforts [e.g., NFL Head, Neck, and Spine Committee (2018a,b)].

### Return to Play Over Time

Time-based factors appear to play a role in the amount of time-off a player receives following a concussion. Interestingly, while Kumar et al. (2014) found that NFL players who sustained a concussion later in the season were less likely to miss any games, the present study did not find any difference in the number of games missed based on the week of the season during which a concussive injury occurred, which could be indicative of the NFL increasing its focus on player health outcomes after a concussion. Additionally, similar to findings in the NBA (Padaki et al., 2016) and NHL (Wennberg and Tator, 2008), results from this study show that the number of games players miss after a concussion has increased each year. While increased time-off could be the result of increasing severity of concussive injuries (Casson et al., 2010), this could also be the result of increased attention from the media on the dangers of concussions (Martin et al., 2017) as well as increased efforts of the NFL to improve player safety [e.g., NFL Head, Neck, and Spine Committee (2018a,b)].

## Conclusion

The results of this study indicate that NFL players miss more games if they (a) are quarterbacks, (b) are involved in more plays, or (c) sustained multiple concussions. This indicates that both player value and health are determinants of time-off after a concussion. Moreover, this also indicates that some players may be less likely to fully recover from a concussion based on non-health-related factors. Nevertheless, the number of games missed following a concussion have increased over time, which could be the result of increased media attention and NFL efforts to increase player safety.

Findings from this study could impact NFL administrators and medical personnel by identifying athletes who are at risk for premature return to play after a concussion and by highlighting factors other than health that impact a player's time-off. This could help to better enforce concussion protocols by ensuring that all athletes receive full treatment and adequate recovery time before returning to play and that proper return-to-play procedures are being followed. Likewise, NFL players can benefit from these findings by knowing what factors determine their time-off after a concussion and help them identify if they are at risk for premature return to play. Knowing these determinants can help players ensure they are treated fairly after sustaining a concussion and allow them to voice their concerns through various outlets (e.g., mediation and collective bargaining). Finally, findings from this study can help increase awareness in NFL team owners and coaches to increase the protection of all players after a concussion, not just highly valued players such as quarterbacks.

### Limitations

This study did not account for the severity of reported concussions or individual differences in responses to concussions, both of which impact recovery time (Hinton-Bayre and Geffen, 2001) and therefore should impact the number of games missed. It is possible that the nature of the quarterback position makes them more susceptible to more severe concussions, thus causing them to require more time following concussions. This study also only examined 3 years of data and does not account for the most recent NFL seasons. Furthermore, this study used data from *PBS Frontline's Concussion Watch*, which only includes reported concussions. With the pressure of masculine stereotypes, NFL players may face scrutiny for admitting injuries, leading to unreported concussions (Anderson and Kian, 2012). Further, 56% of NFL players admit to hiding a concussion to continue playing (Reams et al., 2017), meaning that many concussions occur that are never reported. Therefore, there are likely more concussions that occurred during the sample period but were excluded from the data because they were unreported. However, this lack of reporting, and subsequent lack of time-off following concussions for some players, further emphasizes the existence of disparities between players receiving adequate time-off following concussions.

### Directions for Future Research

There are several paths of future research on concussions and games missed in the NFL. First, interviewing coaches and managers could provide insight as to why quarterbacks receive more time-off after a concussion compared to other players on the team and whether they are considered most valuable. Additionally, more factors should be considered when examining determinants of time-off, including the severity of the concussion and individual responses to the injury. The symptoms and duration of symptoms experienced by each player could be used to scale individual concussions by severity, providing insight into whether the time-off a player was given was the result of health reasons or player value. The prevalence, severity, and effects of unreported concussions should be considered to fully understand the risks and outcomes of all concussions that occur in the NFL and to maximize player safety. Finally, factors of player fame (e.g., media coverage and Twitter followers) could be examined in conjunction with time-off after injury to better understand the role of fans and public perceptions on time-off and player recovery.

## Data Availability Statement

Publicly available datasets were analyzed in this study. This data can be found at: https://www.pbs.org/wgbh/frontline/interactive/concussion-watch/#positions_2015.

## Author Contributions

EH: wrote final draft. LB: wrote second draft. AL: some data collection and wrote initial draft. JF: idea origination, wrote method, and results sections. All authors contributed to the article and approved the submitted version.

## Conflict of Interest

The authors declare that the research was conducted in the absence of any commercial or financial relationships that could be construed as a potential conflict of interest.
